# Memory Foam Pillow as an Intervention in Obstructive Sleep Apnea Syndrome: A Preliminary Randomized Study

**DOI:** 10.3389/fmed.2022.842224

**Published:** 2022-03-09

**Authors:** Vasileios T. Stavrou, Yiannis Koutedakis, Kyriaki Astara, George D. Vavougios, Eirini Papayianni, Ilias T. Stavrou, Fotini Bardaka, Chaido Pastaka, Konstantinos I. Gourgoulianis

**Affiliations:** ^1^Laboratory of Cardio-Pulmonary Testing and Pulmonary Rehabilitation, Department of Respiratory Medicine, Faculty of Medicine, University of Thessaly, Larissa, Greece; ^2^School of Physical Education and Sports Sciences, University of Thessaly, Trikala, Greece; ^3^Institute of Sport, Faculty of Education Health and Wellbeing, University of Wolverhampton, Walsall, United Kingdom; ^4^Department of Neurology, Faculty of Medicine, University of Cyprus, Lefkosia, Cyprus; ^5^Department of Respiratory Medicine, Faculty of Medicine, University of Thessaly, Larissa, Greece

**Keywords:** pillow, memory foam pillow, sleep quality, snoring, desaturation

## Abstract

Specific pillow use is a seldom studied or controlled factor in the setting of sleep disordered breathing. The aim of this study was to investigate the effect of different pillows [own pillow (OP), memory foam pillow (MFP), generic laboratory pillow (LP)] on polysomnography (PSG)-derived parameters in patients with Obstructive Sleep Apnea Syndrome (OSAS). Thirty-two consecutive patients with OSAS were randomly allocated into two groups with randomized pillow usage [Group A: 3 h with LP and 3 h with OP (Age: 53.8 ± 12.5 years, BMI: 32.1 ± 4.6 kg/m^2^); Group B: 3 h with LP and 3 h with MFP (Age: 52.0 ± 6.3 years, BMI: 30.6 ± 2.2 kg/m^2^)]. Statistically significant differences between pillow types were detected in desaturation index and heart rate. In Group B (with MFP), a statistically significant decrease of 47.0 ± 15.9% was observed in snoring events (*p* < 0.05) and 10.6 ± 6.7% in their duration (*p* < 0.05) compared to LP. On the other hand, group A with OP recorded a decrease of 29.1 ± 32.1% in snoring events and 32.5 ± 33.1% in duration, but these values were not statistically significant (*p* > 0.05) compared to LP. These findings indicate that pillow type and usage, often uncontrolled in OSAS studies (contribution to the field), may impact several PSG parameters and are related to a snoring subtype of the syndrome. Secondly, they indicate that a focus on the treatment of the snoring OSAS subtype warrants further dedicated investigation.

## Introduction

Pillows affect sleep quality by maintaining the natural curvature of the spine, thus ensuring optimal sleep posture (1). In patients with mild sleep-disordered breathing, optimal pillow usage may reduce snoring and improves sleep quality efficiency and, by extent, depth (2). Conversely, incorrect pillow placement, such as that occurs during travel, may be detrimental to sleep health (3). Considering Obstructive Sleep Apnea Syndrome's (OSAS's) heterogeneity (4) and the implication of other overlapping symptoms, such as snoring (5), it is necessary to assess the biological context of potential interventions.

Previous studies on pillow usage are limited in number and focus on the role of cervical positional therapy, collectively reporting an improvement on snoring (6–9). Previous research has suggested that the use of custom fitted pillows may represent an efficacious and cost-effective treatment option in mild to moderate OSAS (10), filling a niche where continuous positive airway pressure (CPAP) would not be indicated (11). Considering that pillow design can be guided (12, 13) to alleviate specific symptoms, an evaluation of pillow type impact on sleep health may be directly relevant to their efficacious implementation.

In this context, two major research questions regarding the implementation of specific pillows as a possible OSAS treatment arise: who will benefit and how? As a heterogeneous disease, OSAS' treatment goals extend from severity to phenomenology (4, 14). Toward this end, the effect of pillow usage should be evaluated in order to determine how it shapes polysomnography (PSG)-captured parameters and its implication in sleep disordered breathing phenotypes.

The aim of our study was to investigate the effect of different pillows [own pillow (OP), memory foam pillow (MFP), generic laboratory pillow (LP)] on PSG parameters in patients with OSAS.

## Materials and Methods

### Study Population

This was a single-center, randomized prospective study. Participants were recruited from the province of Thessaly region (Greece). The inclusion criteria were as follows: patients referred for potential sleep disordered breathing following a polysomnography study, an apnea-hypopnea index (AHI) of ≥5 events/h with LP, age between 20 and 80 years, BMI < 40 kg/m^2^, waist to hip ratio <1, and neck circumferences <40 cm. Exclusion criteria were as follows: neurological and psychiatric disorders, musculoskeletal disorders, and awakening during changing pillows.

The study was approved by the Institutional Ethics Committee of the University of Thessaly, Greece (No. 21/09-01-2017) and informed consents were obtained from all participants, in accordance with the Helsinki declaration.

### Full Night Polysomnography

Overnight PSG was performed in accordance with the American Academy of Sleep Medicine (AASM) guidelines (15). PSG-included electroencephalography, electrooculography, submental electromyography, anterior tibialis electromyography, nasal cannula airflow signal using a nasal cannula/pressure transducer system, oral thermistor, electrocardiography, and body position (16). Respiratory efforts were monitored with abdominal and thoracic bands. Arterial SaO_2_ was measured using SpO_2_. Apnea was defined as complete cessation of airflow for at least 10 s in duration. Hypopnea was defined as one of the following three: (1) >50% reduction in airflow, (2) <50% reduction in airflow associated with a desaturation of >3%, or (3) a moderate reduction in airflow with associated arousal by electroencephalography. Apneas were classified as obstructive, central, or mixed according to the presence or absence of respiratory efforts. Snoring was measured by a microphone. Patients with predominant obstructive sleep apneas and AHI ≥ 5 events/h were diagnosed with the OSAS. PSG was performed at the Laboratory of Sleep Disorders (Department of Respiratory Medicine, Faculty of Medicine, University of Thessaly, Greece) using an Alice^®^4 computer system (Philips Respironics, PA, USA). PSG scoring was performed by specialists blinded to pillow usage and group allocation.

### Learning Effect Attenuation

In an attempt to eliminate any PSG learning effect, volunteers were randomly divided into two groups, (Group A: 3 h with LP and 3 h with their OP; Group B: 3 h with LP and 3 h with MFP). The replacement of the pillow during PSG was (a) conducted by a sleep technician in a randomized order and (b) at the awaking phase.

### Epworth Sleep Scale Questionnaire

Sleepiness was assessed using the validated Greek version of the Epworth Sleepiness Scale (ESS), (17) a self-administered questionnaire evaluating the possibility of dozing in a variety of situations (18).

### Pillow Characteristics

The dimensions of the LPs were 12.0 × 60.0 × 36.0 cm (Height × Length × Width) and was made with microfiber. The pillows owned by patients had variant dimensions (14.2 ± 0.8 × 55.0 ± 3.4 × 44.2 ± 3.6 cm; Height x Length × Width) and were made of polyester (34%), foam shredded (29%), and goose feather (37%). The MFPs had dimensions of 16.0 × 70.0 × 40.0 cm (Height × Length × Width) and was made of memory foam with aloe vera extract (Media Strome^®^, Greece).

### Statistical Analyses

Pairwise comparisons per group (Group A: LP vs. OP and Group B: LP vs. MFP) were performed with the dependent samples *t*-test. We calculated the standardized rates of change between sequential measurements via the formula [(Post-Pre)/Pre], where “post” refers to post-intervention and “pre” refers to pre-intervention. These rates were compared between intervention groups via the independent Samples *t*-test. Data are presented as absolute numbers, percentages, or mean values ±Sd where applicable. For all statistical analyses, a *p*-value < 0.05 was considered statistically significant. All analyses were performed via the IBM SPSS Statistics 25 (SPSS Inc., Chicago, IL, USA) software package.

## Results

**Figure 1:**
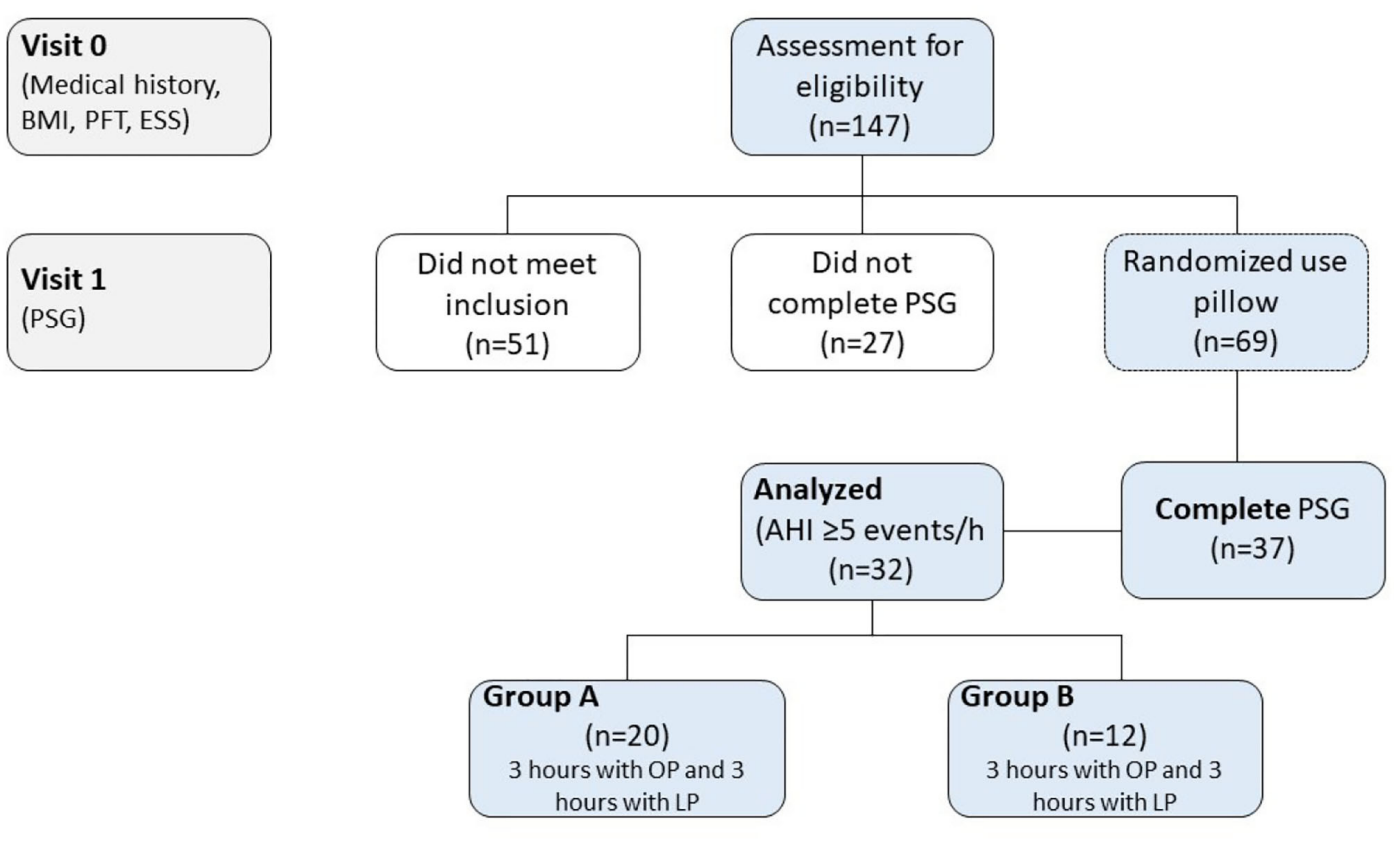
Study flow diagram. BMI, body mass index; ESS, Epworth sleepiness scale; LP, laboratory pillow; MFP, memory foam pillow; OP, own-pillow; PFT, pulmonary function test; PSG, polysomnography study.

Out of the 147 individuals who were assessed for eligibility, 32 were included in the study (Figure 1). Demographic characteristics were not significantly different between groups (Table 1). Table 2 illustrates statistical differences in Heart Rate (HR) values between two types of pillows (LP vs. MFP) in awakening phase (*p* = 0.003), REM stage (*p* = 0.003), and non-REM stage (*p* = 0.003). The group with the MFP pillow showed higher values in HR during awakening phase and non-REM stage compared to the group with OP (*p* < 0.05). There were statistically significant differences between pillow types and the standardized rate of change for the arousal index and the number of snoring events (*p* < 0.05). Table 2 provides information on snoring duration and snoring events during PSG. Unlike Group A which demonstrated no statistical differences in snoring parameters, Group B recorded significantly lower number of snoring events by 47.0 ± 15.9 % (106.0 ± 17.8 vs. 55.8 ± 23.7 events, *p* = 0.002) and snoring duration by 10.6 ± 6.7% (42.2 ± 5.0 vs. 37.5 ± 3.7 min, *p* = 0.002) with MFP compared to LP.

**Table 1:** Patient characteristics.

	**Total** **(*n =* 32)**	**Group A** **(*n =* 20)**	**Group B** **(*n =* 12)**	***P* value**
Age, yrs	53.1 ± 10.5	53.8 ± 12.5	52.0 ± 6.3	0.685
Body mass index, kg/m^2^	31.6 ± 3.9	32.1 ± 4.6	30.6 ± 2.2	0.287
Gender, (Male/Female)	24/8	16/4	8/4	0.272
Smokers, %	31	35	25	0.569
Hypercholesterolemia, %	38	45	25	-
Gastroesophageal reflux disease, %	6	10	-	-
Hypertension (Stage I), %	6	10	-	-
Asthma, %	6	-	17	-
Comorbidity-free, %	44	35	58	-
Epworth sleepiness scale, score	9.3 ± 4.2	9.2 ± 4.6	9.3 ± 3.7	0.656

*Data are expressed as mean ± standard deviation or percentages*.

**Table 2:** Polysomnography parameters results.

	**Total (*n =* 32)**	**Group A (*n =* 20)**			**Group B (*n =* 12)**			***P* value between groups**
		**LP**	**OP**	***P value***	**LP**	**MFP**	***P* value**	
AHI, events/h	34.9 ± 22.6	39.0 ± 27.7	42.2 ± 32.5	0.279	28.0 ± 5.9	26.6 ± 9.0	0.301	0.173
Non-REM, %	34.8 ± 24.3	40.2 ± 29.1	41.5 ± 33.8	0.514	26.0 ± 8.4	27.0 ± 10.6	0.340	0.498
REM, %	40.8 ± 22.5	38.3 ± 24.6	44.3 ± 26.8	0.247	44.9 ± 18.9	35.1 ± 15.4	0.032	0.946
Body position distribution desaturation at								
Left, %	31.1 ± 34.2	28.0 ± 11.4	16.7 ± 8.6	0.001	68.9 ± 27.4	46.2 ± 29.3	0.013	<0.001
Right, %	51.9 ± 43.8	47.2 ± 54.3	14.3 ± 5.1	0.001	59.8 ± 14.3	59.5 ± 21.3	0.875	0.934
Up, %	51.3 ± 68.4	67.1 ± 83.2	83.4 ± 88.1	0.274	25.0 ± 5.5	6.5 ± 3.1	0.005	0.145
Averages values in HR during awakening, bpm	77.1 ± 10.2	75.2 ± 12.0	78.5 ± 22.9	0.247	80.4 ± 4.0	84.6 ± 5.2	0.003	0.799
Averages values in HR non-REM stage, bpm	66.6 ± 9.3	68.0 ± 8.3	69.4 ± 7.7	0.305	64.3 ± 10.8	72.2 ± 4.7	0.003	0.051
Averages values in HR REM stage, bpm	72.1 ± 10.2	69.8 ± 11.7	72.1 ± 16.3	0.191	75.8 ± 6.0	80.3 ± 9.1	0.003	0.459
Desaturation index during sleep	34.8 ± 25.0	40.3 ± 29.9	43.7 ± 35.3	0.296	25.6 ± 8.9	23.3 ± 8.6	0.017	0.140
Desaturation duration, min	14.4 ± 9.9	16.0 ± 12.5	16.3 ± 12.9	0.747	11.8 ± 3.5	10.9 ± 3.8	0.054	0.253
Averages values in SaO_2_ per respiratory event, %	90.0 ± 3.8	89.6 ± 4.7	88.7 ± 4.9	0.038	90.8 ± 1.2	90.7 ± 1.1	0.011	0.143
Minimum SaO_2_ per respiratory event, %	83.0 ± 7.2	83.2 ± 8.7	81.6 ± 8.8	0.192	82.7 ± 3.7	84.8 ± 2.9	0.006	0.159
Awakening index, n/h	37.5 ± 24.6	41.5 ± 30.1	53.9 ± 29.8	<0.001	31.0 ± 7.9	30.0 ± 10.0	0.301	0.031
Snoring duration, min	36.8 ± 21.2	33.6 ± 26.2	30.2 ± 17.2	0.911	42.2 ± 5.0	37.5 ± 3.7	0.002	0.356
Snoring events	100.3 ± 48.8	96.8 ± 60.6	94.5 ± 55.6	0.852	106.0 ± 17.8	55.8 ± 23.7	0.002	0.058

*Data are expressed as mean ± standard deviation or percentages changes. AHI, apnea-hypopnea index; HR, heart rate; Left: Desaturation during sleep in left body position; LP, laboratory pillow; MFP, memory foam pillow; OP, own-pillow; REM, rapid eye movement during sleep; Right: Desaturation during sleep in right body position; Up: Desaturation during sleep in supine body position*.

## Discussion

Our study aimed to investigate the effect of pillow type (OP and/or MFP vs. LP) in patients with OSAS and its effects on polysomnographic and phenotypic parameters of the syndrome. We report on preliminary results from our ongoing study, indicating that MFP improved several PSG-captured parameters in patients with OSAS, mainly the snoring phenotype. Reduction of quantity and intensity of snoring events, along with improved oxygenation relative to body position, were among the significant PSG-captured effects of MFP usage. These findings indicate that pillow type and usage, often uncontrolled in OSAS studies, impacts several PSG parameters and is related to a snoring subtype of the syndrome. Secondly, they indicate that a focus on the treatment of the snoring OSAS subtype warrants further, dedicated investigation.

In general, the supine position has been implicated in the exacerbation of apneic episodes in OSAS (19), whereas prone position may be alleviating (20). Our findings indicate that this alleviation may be captured by the improvement of desaturation index and snoring events, and thus may be more relevant to the snoring phenotype (21).

The use of different pillow types showed HR changes in both groups of patients with OSAS. However, the MFP sub-group showed higher values in HR around apneic episodes as recorded by awakening phase and minimum SaO_2_ per respiratory event compared to the OP sub-group. Before apneic episodes, bradycardia occurs, followed by HR increase that may or may not produce cortical arousals (22). The lower the HR variability, the lower the arousals that occur, leading to less sleep fragmentation and sleep quality. OSAS severity is associated with the abnormal adaptability of the autonomic nervous system (ANS), and this association is reversible up to an extent as the treatment of OSAS is implemented (23). The application of a more suitable pillow could reinforce the therapeutic effects.

The characteristics of snoring were also significantly ameliorated when the MFP was applied. Snoring is elicited by a Bernoulli effect on the soft palate, as negative pressure induces its vibration during an obstructive respiratory event (24). Maintaining airway patency via positional therapy could therefore be expected to reduce snoring events. The use of “smart” pillows for positional therapy in OSAS have reported a reduction of snoring events and overall better sleep quality (6) – findings that are similar to our own.

Positional therapy in OSAS phenotypes where obstructive events and snoring are central features has been previously proposed as a simple and effective intervention (25). Similarly, pillows designed to address cervical positioning and, by extension, airway patency have been associated with a reduction of snoring events and duration (7, 10).

### Limitations

It is reasonable to assume that the present study might have been influenced by methodological limitations such as the lack of information on allergenic effects of synthetic pillows (26), although such effects were not diagnosed or reported in our study. Furthermore, longer follow-up studies are needed in order to determine whether the effects noted in our study are sustained. Another important limitation is that measurements of the mandibular plane-hyoid distance, the small posterior airway space diameters, and tongue volume were not included in our study (27). Finally, the production of groups via randomization process may have created groups with the potential to exhibit greater improvement following the implementation of different pillow types. This has been addressed with both between and within-group subject comparisons in order to simultaneously assess the effect of pillow placement and different pillow type. The randomization involved patient allocation rather than pillow placement post-allocation. In order to detect sequence effects, we would have to deploy a different design, i.e., MFP-LP-MFP vs. LP-MFP-LP, and then determine sequences within and then across groups. We did not deploy this aforementioned design on the premises of introducing additional disruption in the PSG that would correspondingly need to be accounted for.

## Conclusions

Compared to either own or regular LPs, MFP seems to be more effective in decreasing snoring events and snoring duration. As a relatively easy to implement intervention, large studies should focus on the role of appropriate pillows as an adjunctive treatment modality of OSAS. Furthermore, our study indicates that the usage of the patient's OP rather than LPs may give a closer-to-life laboratory investigation of OSAS.

## Data Availability Statement

The raw data supporting the conclusions of this article will be made available by the authors, without undue reservation.

## Ethics Statement

The studies involving human participants were reviewed and approved by Institutional Ethics Committee of the University of Thessaly, Greece, No. 21/09-01-2017. The patients/participants provided their written informed consent to participate in this study.

## Author Contributions

VTS, YK, and KIG conceived and designed the experiments. VTS, GDV, EP, and FB analyzed the data. VTS, YK, KA, IS, and GDV wrote the paper. VTS, YK, GDV, and KIG edited the paper. All authors contributed to the article and approved the submitted version.

## Conflict of Interest

The authors declare that the research was conducted in the absence of any commercial or financial relationships that could be construed as a potential conflict of interest.

## Publisher's Note

All claims expressed in this article are solely those of the authors and do not necessarily represent those of their affiliated organizations, or those of the publisher, the editors and the reviewers. Any product that may be evaluated in this article, or claim that may be made by its manufacturer, is not guaranteed or endorsed by the publisher.
